# Ischemic stroke as the first manifestation of Takayasu arteritis: high resolution magnetic resonance imaging

**DOI:** 10.1007/s10072-021-05552-0

**Published:** 2021-08-12

**Authors:** Zhiming Kang, Zhipeng Xu, Xiangbo Wu, Chuang Nie, Jiaqi Yin, Bin Mei

**Affiliations:** Neurology Department, Zhongnan Hospital of Wuhan University, Wuhan, 430071 China

To the Editor,

Takayasu arteritis (TA) is a chronic non-specific inflammatory arteritis that primarily affects the aorta and its main branches. Ischemic stroke, a less common complication of the disease, can sometimes be the first manifestation of TA. We report a case that was first diagnosed with Takayasu arteritis for ischemic stroke, which is rarely reported before. When assessing the disease activity, we tested serological inflammatory biomarkers including erythrocyte sedimentation rate (ESR) and C-reactive protein (CRP), which were within normal reference range. Thus, the patient underwent a diagnostic evaluation with high-resolution magnetic resonance imaging (HR-MRI), which suggested active TA. Then, the patient received immunosuppression therapy. She recovered well from stroke and did not have a recurrence of ischemic events.

## Case presentation

**Fig. 1:**
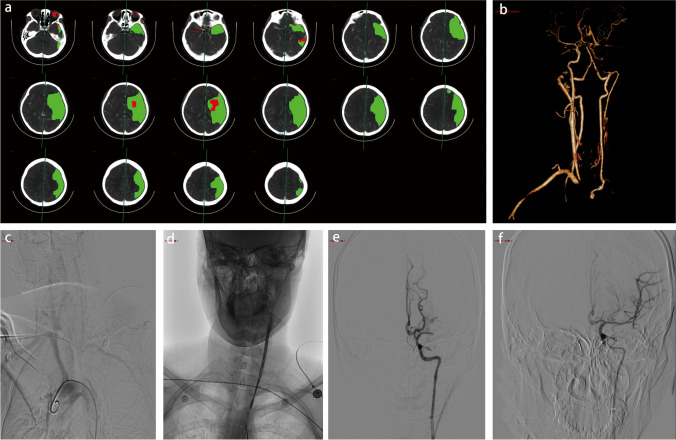
The CTP (**a**), CTA (**b**), and DSA (**c–f**) images. **a** The CTP images showed a mass of salvageable brain tissue in the left MCA territory. **b** The reconstructed CTA images showed occlusion of the left SCA, left ICA and M1 segment of the left MCA, and stenosis of the left CCA. **c** Angiography of the aortic arch showed occlusion of the proximal left SCA. **d** Angiography of the left CA showed stenosis of the left CCA and occlusion of the left ICA. **e** Angiography failed to showed the left MCA from the M1 segment. **f** Full reperfusion of the MCA was achieved after mechanical thrombectomy

A 27-year-old woman without traditional vascular risk factors and family history presented with sudden onset of right-side hemiparesis and aphasia for twenty minutes. Neurological examination showed global aphasia, right-side partial facial weakness, and severe right hemiparesis. Her National Institutes of Health Stroke Scale (NIHSS) score was 11. A no-contrast compute tomography (CT) of the brain was negative for acute hemorrhage, and she received intravenous alteplase with a standard dose of 0.9 mg/kg. CT angiography (CTA) showed an occlusion of the proximal left subclavian artery (SCA), left carotid artery (CA), and M1 segment of the left middle cerebral artery (MCA) (Fig. 1b). CT perfusion (CTP) demonstrated a mismatch ratio of 21.1 with the infarct core volume of 7.4 ml and the ischemic penumbra of 155.8 ml (Fig. 1a). As her symptoms persisted with a mass of salvageable brain tissue, balloon dilatation of the left CA and mechanical thrombectomy of the left MCA was performed with reperfusion flow of the left MCA from thrombolysis in cerebral infarction (TICI) 0 to TICI 3. Digital subtraction angiography (DSA) before the procedure was consistent with CTA, and the diagnosis of TA was suspected (Fig. 1c–f). Then, she was admitted to our stroke unit for further management.

**Fig. 2:**
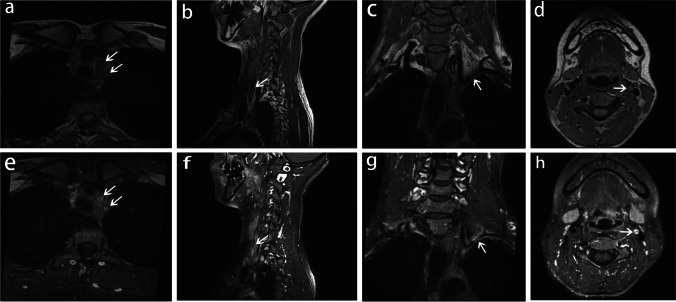
High-resolution MR images acquired from a clinical 3.0 Tesla system. T1-weighted spin-echo images (**a-d**) revealed vascular wall thickening, while gadolinium contrast-enhanced images (**e–h**) showed smooth concentric mural enhancement. (**a**) and (**e**), (**b**) and (**f**), (**c**) and (**g**), and (**d**) and (**h**) displayed the left CCA and the proximal left SCA in axial view, the left CCA in sagittal view, the left SCA in coronal view, and the left ICA in axial view, respectively

To confirm the diagnosis and evaluate the activity of TA, inflammatory biomarkers such as ESR, CRP, and interleukin-6 were tested, but all were within normal reference range. Additional workup, including B-type natriuretic peptide, biomarkers of myocardial injury, hemoglobin A1c, low-density lipoprotein, autoimmune antibodies, and electrocardiogram showed no obvious abnormality. Then, HR-MRI of the whole brain was performed, which demonstrated obvious vascular wall thickening of the left common CA (CCA), the proximal left SCA, and the left internal CA (ICA) (Fig. 2a–d). Contrast-enhanced T1-weighted imaging revealed marked concentric enhancement of the thickened vascular walls, suggesting the activity of TA (Fig. 2e–h). The patient received immunosuppression drugs including metacortandracin and methotrexate. She had no clinically significant disability with modified Rankin score of 1 during the follow-up.

## Discussion

TA, a chronic non-specific inflammatory disease of unknown etiology, is most prevalent in Asian female. Immune-mediated vascular inflammation leads to vessel wall fibrosis, causing symptoms of organ ischemia [1]. Neurologic symptoms of TA include headache, dizziness, visual disturbances, TIA, and stroke. Ischemic stroke or TIA is a less common complication of TA, with an estimated prevalence of 15.8% according to a meta-analysis [2]. However, it could be the first manifestation in some cases. Our patient was first diagnosed with TA for ischemic stroke. Therefore, it is important to consider TA in young stroke patients without traditional risk factors or family history of stroke, and a full diagnostic evaluation of stroke etiologies is needed.

Angiography has been proposed as the gold standard for the diagnosis of TA. TA is featured with stenosis or occlusion or aneurysm formation of the aorta and its primary branches in angiographic imaging [1]. However, angiography is an invasive method and is unable to reveal the wall information of involved vessels. Neuroimaging technology has advanced in recent years, and HR-MRI can now be used to evaluate the morphology of extra- and intracranial vascular walls [3]. The technology is a noninvasive and radiation-free method with equal sensitivity and specificity with angiography in diagnose TA. Besides, HR-MRI can reveal both the vessel wall and lumen of artery simultaneously with good spatial resolution by using the black blood and three-dimensional technique, which is essential for the evaluation of TA. The HR–MR characteristic of TA is a smooth crescent-like or concentric wall thickening with enhancement in active individuals. Arterial involvement is more common in single lesions that develop in multiple arteries than multiple lesions that develop in a single artery. It is different from atherosclerosis that featured with rough eccentric wall thickening and irregular plaques [3]. The HR-MRI of our patient showed smooth concentric wall thickening with obvious enhancement in the left common carotid artery, the proximal left subclavian artery, and the left internal carotid artery, confirming the diagnosis of TA.

In addition, HR-MRI may be an alternative way to assess the activity of TA since no reliable serological marker has been identified until now. ESR was previously used as an active biomarker, but it has been observed to raise in nearly half of the patients in clinical remission and remain normal in 28% of the patients with active disease, indicating a poor specificity and sensitivity [4]. Mural thickening and intramural contrast uptake on HR-MRI has been confirmed as an indication of inflammation of the vessel wall by biopsy in giant cell arteritis [5]. Therefore, we speculate that mural thickening and wall enhancement in TA are signs of disease activity and successful immunosuppression therapy confirmed our hypothesis.

In conclusion, ischemic stroke can be the first manifestation of TA, especially in young patients. HR-MRI is a reliable technology for the evaluation of TA, and further exploration is needed.

## Data Availability

Not applicable.
